# Susceptibility-Weighted Imaging for the Noncontrast Evaluation of Hepatocellular Carcinoma: A Prospective Study with Histopathologic Correlation

**DOI:** 10.1371/journal.pone.0098303

**Published:** 2014-05-30

**Authors:** Wei Chen, Zachary DelProposto, Wei Liu, Mohammad Kassir, Zhiyuan Wang, Jun Zhao, Bing Xie, Yaming Wen, Jian Wang, Jiani Hu

**Affiliations:** 1 Department of Radiology, Southwest Hospital, Third Military Medical University, Chongqing, China; 2 Department of Radiology, Henry Ford Hospital, Detroit, Michigan, United States of America; 3 Department of Hepatobiliary Surgery, Southwest Hospital, Third Military Medical University, Chongqing, China; 4 Department of Radiology, Wayne State University, Detroit, Michigan, United States of America; 5 Cancer Biotherapy Center, Hunan Provincial Tumor Hospital, Xiangya Medical School, Central South University, Hunan, China; Yonsei University College of Medicine, Korea, Republic Of

## Abstract

**Background:**

Specific morphologic features of hepatocellular carcinoma (HCC) on imaging have identifiable pathologic correlates as well as implications for altering surgical management and defining prognosis. In this study, we compared susceptibility-weighted imaging (SWI) to conventional techniques and correlated our findings with histopathology to determine the role of SWI in assessing morphologic features of HCC without using a contrast agent.

**Methods:**

86 consecutive patients with suspected HCC were imaged with MRI (including T1, T2, T2*, and SWI) and subsequently CT. 59 histologically-proven HCC lesions were identified in 53 patients. Each lesion on each imaging sequence was evaluated by two radiologists, and classified with respect to lesion morphology, signal intensity relative to surrounding hepatic parenchyma, presence of a pseudocapsule, presence of venous invasion, and internal homogeneity.

**Results:**

Histopathology confirmed pseudocapsules in 41/59 lesions. SWI was able to detect a pseudocapsule in 34/41 lesions; compared to conventional T1/T2 imaging (12/41) and T2* (27/41). Mosaic pattern was identified in 25/59 lesions by histopathology; SWI confirmed this in all 25 lesions, compared to T1/T2 imaging (13/25) or T2* (18/25). Hemorrhage was confirmed by histopathology in 43/59 lesions, and visible on SWI in 41/43 lesions, compared to T1/T2 (7/43) and T2* (38/43). Venous invasion was confirmed by histopathology in 31/59 patients; SWI demonstrated invasion in 28/31 patients, compared to T1/T2 (7/31) and T2* (24/31).

**Conclusions:**

SWI is better at identifying certain morphologic features such as pseudocapsule and hemorrhage than conventional MRI without using a contrast agent in HCC patients.

## Background

Hepatocellular carcinoma (HCC) is the fifth most common cancer worldwide, primarily occurring in patients with chronic liver disease [1]. Advances in magnetic resonance imaging (MRI) continue to improve the diagnostic accuracy for HCC detection. Contrast-enhanced MRI, the most widely used clinical method to detect HCC, relies on differences in perfusion dynamics between normal hepatic parenchyma and that of altered tumor vascularity, which consists of unpaired arteries and sinusoidal capillarization. Presently, few imaging options are available in patients with poor renal function, since poor renal function precludes the use of gadolinium-based contrast enhancement [2]. Therefore, detection of HCC by noncontrast MRI relies primarily on lesion morphology. Characteristic morphologic features of HCC, detectable by both histopathology and MRI, are a fibrous capsule and a mosaic pattern of contrast enhancement on MRI (corresponding to fibrous septa and macroscopic heterogeneity, respectively, on histopathology) [3], [4]. Among HCC lesions larger than 2 cm, 60–80% have fibrous capsules [5]. The presence of a fibrous capsule is considered a favorable prognostic factor, allowing surgical cure of HCC [6] by providing a simpler resection with a greater preservation of surrounding non-malignant liver parenchyma, which is particularly important for patients with decreased liver function [7]. The mosaic pattern is found in up to 63% of cases of HCC [8], [9], and represents areas of growth intermixed with areas of necrosis and regenerative liver tissue [4], [5]. Additionally, it has been found that the presence of vascular invasion (particularly microvascular invasion) is an important prognostic factors in HCC [10].

Susceptibility Weighted Imaging (SWI) is an emerging tool that utilizes magnetic susceptibility differences between tissues to enhance the detection of hemorrhage. So far, SWI has been predominantly confined to neuroimaging applications, where it has demonstrated superior sensitivity in detecting lesions with microhemorrhage, calcification or changes in oxygenation level when compared to other imaging techniques [11]–[13]. The successful application of 3D SWI in abdominal imaging has faced technical challenges, primarily due to the long acquisition time, leading to substantial motion artifact from respiration and bowel peristalsis [14]. A recently developed multi-breath-hold two dimensional (2D) GRE-based SWI sequence (Work-in-progress sequence #608, Siemens Healthcare) has been used to evaluate siderotic nodules in cirrhotic livers, and to accurately detect intratumoral hemorrhage (in renal cell carcinoma and HCC) and venous vascularity within HCC [15]–[19]. HCC typically exhibit a heterogeneous appearance on MRI, depending upon morphologic changes in the tumor (such as necrosis, hemorrhage, and fatty change). However, some benign liver lesions occasionally undergo necrosis and intratumoral hemorrhage and therefore contrast-enhanced MR imaging is the preferred and most commonly used method for diagnosing HCC. Intratumoral hemorrhage has been associated with HCC expansion and rupture following therapy, and delineation of hemorrhage (and subsequent avoidance when using region-of-interest measurements) may improve ADC measurements [17], [20]. In patients with poor renal function, the use of gadolinium chelates poses a significant risk, necessitating the evaluation of new MR imaging techniques [2]. SWI can provide more detailed information than conventional liver MRI with respect to the evaluation of tumor boundaries, blood products, and venous vasculature [19]. SWI may allow the differentiation of hypervascular liver lesions, particularly in patients who cannot receive gadolinium contrast [15].

The goal of this study was to compare the imaging manifestations of hepatocellular carcinoma with SWI to those of conventional non-contrast enhanced liver MRI. Specifically, we evaluate the ability of SWI to detect tumor pseudocapsule, mosaic pattern, intratumoral hemorrhage, and macrovascular invasion, to better investigate the diagnostic potential of SWI for the detection of hepatocellular carcinoma.

## Methods

### Patient Population

The study was approved by the institutional review board of Southwest Hospital in Chongqing, China, and performed in accordance with the ethical guidelines of the Declaration of Helsinki. After a thorough explanation of the study to the patient, written informed consent was obtained. From December 2010 through August 2011, 86 consecutive patients with suspected HCC underwent hepatic MRI. Patient were included in the study if (a) features suspicious for HCC were detected on MRI, in combination with clinical history and elevated serum AFP level (>400 ng/ml) and (b) diagnosis of HCC confirmed by means of histology. Among the 86 patients, 53 patients (62%) were ultimately diagnosed with histologically-proven HCC, with a total of 59 lesions identified (49 patients had a single lesion, 3 patients had 2 lesions, and 1 patient had 5 lesions). Patients underwent either hepatic lobar and/or segmental resection (n = 52) or orthotopic liver transplantation (n = 1) at our institution. All patients had been referred for subsequent MR imaging for evaluation of liver and biliary tract. Of the 53 patients, there were 31 men and 22 women (age range 25–71 years; mean age 51 years). Forty-six of the 53 patients had evidence of hepatic viral infection (hepatitis B, n = 43; or hepatitis C, n = 3). The interval between the date of MRI and surgery ranged from 2 to 18 days (mean, 4.36±3.21 days).

### Computed Tomographic Imaging

All subjects were imaged with dual-source CT (Definition, Siemens Healthcare, Forchheim, Germany). Imaging parameters were: 250 mAs, 120 kVp, 1.2 mm beam collimation with a 0.5 s gantry rotation time. Field of view (FOV) was 35 cm, with a reconstruction thickness and interval both of 5 mm. No oral contrast was administered. The examination consisted of non-contrast images followed by three dynamic images acquired 35 s (hepatic arterial phase), 70 s (portal venous phase), and 180 s (delayed phase) following the intravenous administration of 100–120 ml Ultravist 370 (Bayer-Schering, Leverkusen, Germany) at a rate of 3–4 ml/s.

### Magnetic Resonance Imaging

MR imaging for all subjects was performed on a 3.0 T whole-body system (Magnetom Trio, Siemens Healthcare, Erlangen, Germany) using a standard 12-channel matrix coil. All patients were examined with routine hepatic MR imaging and MRCP. 48 patients were examined with intravenous contrast enhancement. Contrast-enhanced images were obtained after administration of 0.1 mmol/kg gadopentate dimeglumine (Magnevist; Schering, Berlin, Germany) with a T1-weighted fat-suppressed spoiled-gradient echo sequence.

The following MR pulse sequences were used for all patients: transverse T1-weighted 2D gradient echo (GRE) (flip angle 70°, TR/TE 140/2.46 ms), transverse T2-weighted 2D fast spin echo (flip angle 122°, TR/TE = 3700/84 ms, ETL 9), transverse T2*-weighted 2D GRE (flip angle 20°, TR/TE = 150/10 ms) and transverse abdominal 2D SWI (flip angle 20°, TR/TE = 150/10 ms). For all sequences, FOV was 280×285 mm^2^ and matrix was 320–384×250; slice thickness was 5 mm with a gap of 1 mm. Depending upon hepatic size, two to three breath holds of 15 to 20 s duration were used for each sequence.

For abdominal SWI, a recently developed multi-slice 2D GRE sequence with SWI reconstruction was used [15]. An SWI dataset consisted of 3 contiguous 10-slice transverse acquisitions through the liver, with the duration of each acquisition suitable for a single breath-hold (less than 20 s). Automatic SWI postprocessing was performed to produce both magnitude and phase images. Postprocessing consisted of 4 steps: 1) data from each channel was processed through a high-pass filter to remove background artifacts, 2) the magnitude and phase images from each channel were combined to create a final (complex) image, 3) corrected phase images were created from the final complex images, and 4) the magnitude image was multiplied with the normalized phase mask (calculated from each phase-corrected image) to produce the final image.

### Histopathology Evaluation

All specimens were evaluated by members of the department of pathology at our institution. Livers were sectioned into 1 cm slabs, and HCC lesion locations within the liver were recorded. Lesions were measured in the three largest dimensions. Each lesion was then excised for additional histologic analysis.

### Image Evaluation

CT and MR images of all patients were evaluated on a Syngo workstation. CT, T1WI, T2WI, T2*WI and SWI images were reviewed and analyzed by two radiologists, W.C. and J.W. (with 18 and 25 years of hepatic imaging experience, respectively), who were blinded to pathological results. SWI magnitude images were considered equivalent to T2*-weighted gradient echo images during image interpretation.

Lesion signal intensities were visually compared with the surrounding liver and categorized as hyperintense, isointense, or hypointense.

The total number of lesions was recorded. Lesions were assessed for the presence of a capsule, internal appearance (including the presence of intratumoral hemorrhage and mosaic pattern), and the presence of venous obstruction. The capsule was determined to be present on T1-weighted images if a peripherally hypointense band-like structure was evident, and on T2-weighted images if a band-like structure with two different layers (inner hypointense, outer hyperintense) [4], [21]. Tumor venous and/or portal obstruction was defined as distention of the hepatic and/or portal vein lumen thrombus or by the proximity of the tumor and the thrombosed vessel.

Evaluation of lesion internal architecture included the presence of intratumoral hemorrhage, necrosis, fat, and calcifications. Hemorrhage was defined as components which were hyperattenuating to unenhanced liver at CT and hyperintense to the unenhanced liver on T1-weighted MR images. Hemorrhage was divided into 4 grades: grade 0: no dot-like foci of intratumoral hemorrhage signal intensity, grade 1: 1–5 discrete foci, grade 2: 6–10 discrete foci, and grade 3: more than 10 discrete foci. Necrosis was defined as non-enhancing areas with attenuation similar to that of gallbladder contents and as signal hyperintensity on T2-weighted images. Fat was identified when the tumor components had attenuation coefficients lower than those of water and/or bile or urine on non-enhanced CT scans and as signal hypointensity on T1-weighted fat-suppressed MR images or signal loss on opposed-phase gradient-echo MR images. Calcifications were defined as an area of distinctly different attenuation or signal intensity in or near the center of the lesion on unenhanced images or on images obtained at different phases of enhancement.

To determine the signal-intensity characteristics of intratumoral hemorrhage (ITH) on CT, different MR pulse sequences from the same patient were correlated and compared using both qualitative and quantitative methods. The criteria for ITH were as follows: for CT imaging, a hemorrhagic focus was diagnosed if there was hyperdensity within the lesion. For conventional MR sequences, a new hemorrhagic focus was diagnosed if the lesion was hyperintense at T1WI and hyperintense on T2WI and GRE T2*images. A chronic hemorrhagic focus was diagnosed if the lesion was hypointense or isointense on T1WI and hypointense on T2WI and GRE T2*images. Once a hyperintense area was depicted at T1WI, the pre-contrast T1-weighted fat-suppressed spoiled-echo images were reviewed so as to rule out the possibility of intratumoral fatty component. On SWI, hemorrhagic foci were defined as linear, dot-like or patchy hypointensity deemed intratumoral susceptibility signal (ITSS). Upon detecting a suspected hemorrhage, observers reviewed several adjacent slices to exclude the possibility of a vessel in cross-section. Additionally, SWI phase images were also reviewed to differentiate findings from calcifications, as calcifications appear hyperintense on phase images and hypointense on SWI. The confidence scale of hemorrhagic foci was based on whether each lesion represented hemorrhagic foci with scores ranging from 0 to 4 (0 = definitely not present; 1 = probably not present; 2 = indeterminate; 3 = probably present and 4 = definitely present). The sensitivity calculations were based on only those lesions awarded a confidence rating of 3 or 4 score. A final decision was achieved by consensus. A true-positive MR imaging result was defined as the presence of ITH that also had a surgically proved positive result. Contrast-enhanced images were not used for evaluation of internal architecture or intratumoral hemorrhages.

After qualitative analysis, quantitative analysis was performed out for lesions in which intratumoral hemorrhage was identified. The hemorrhage: tumor signal-to-noise ratio (SNR) was then calculated. The signal intensity (SI) of hemorrhagic foci and hepatic parenchyma were taken by using operator-defined regions of interest (ROIs). ROIs were drawn in the same location for each sequence. The hemorrhage-parenchymal SNR was calculated with the following formula: SI of hemorrhage/SI of parenchymal. If there were multiple hemorrhagic foci, the smallest one was chosen to for analysis. SNR of hemorrhage that was 0.7–1 was scored 1; if SNR of hemorrhage was 0.4–0.7 it was scored 2, and if SNR of hemorrhage was less than 0.4, it was scored 3.

### Statistical Analysis

SWI was compared with CT, T1WI, T2WI and T2* sequences. For qualitative analysis, the sensitivity, specificity, and accuracy were assessed. For quantitative analysis, SNR of hemorrhagic foci were compared. To assess the interobserver agreement for the qualitative analysis, Fleiss’ kappa statistic for two observers was calculated. A value of less than 0.200 indicated positive but poor agreement; 0.210–0.400, fair agreement; 0.410–0.600, moderate agreement; 0.610–0.800, good agreement; and greater than 0.810, excellent agreement. The statistical analysis was performed with the Statistical Package for Social Science software (SPSS), Version 16.0. A p value of less than 0.05 was considered to indicate a significant difference.

## Results

A total of 59 hepatocellular carcinomas were detected. With respect to tumor size, 17 lesions were smaller than 3 cm, 38 lesions were 3.1–10 cm, and 4 lesions were greater than 10 cm. 3/59 (5%) lesions were hypointense, 9/59 (15%) were isointense and 47/59 (80%) were hyperintense visually compared with the surrounding liver on T2WI, T2*WI, and SWI images. The interobserver agreement for the qualitative assessment 0.55 (fair) for T1WI, 0.63 (good) for T2WI, 0.79 (good) for T2*WI, and 0.89 (excellent) for SWI. HCC imaging features using different MR imaging sequences are summarized in Table1. The sensitivity, specificity, and accuracy of morphologic features evaluation is detailed in Table 2, with aggregate results detailed in Table 3.

**Table 1:** HCC imaging features using different MR imaging sequences.

Feature	T1WI and T2WI	T2*WI	SWI
Pseudocapsules	12/41	27/41	34/41
Mosaic pattern	13/25	18/25	25/25
Hemorrhage	7/43	38/43	41/43
Venous invasion	7/31	24/31	28/31

**Table 2:** Sensitivity, Specificity, and Accuracy of the morphologic features of hepatocellular carcinoma on noncontrast imaging.

	Sensitivity (%)	Specificity (%)	Accuracy (%)
**Reader 1**			
Pseudocapsule			
T1WI/T2WI	32 (13/41)	33 (6/18)	32 (19/59)
T2*WI	71 (29/41)	50 (9/18)	64 (38/59)
SWI	78 (32/41)	67 (12/18)	75 (44/59)
Mosaic pattern			
T1WI/T2WI	(10/25)	(15/24)	(25/59)
T2*WI	(16/25)	(16/25)	(32/59)
SWI	25/25	28/34	(43/59)
Hemorrhage			
T1WI/T2WI	14 (6/43)	55 (10/18)	27 (16/59)
T2*WI	91 (39/43)	61 (11/18)	64 (38/59)
SWI	93 (40/43)	78 (14/18)	92 (54/59)
Venous invasion			
T1WI/T2WI	23 (7/31)	64 (18/28)	42 (25/59)
T2*WI	71 (22/31)	75 (25/28)	73 (43/59)
SWI	90 (28/31)	(25/28)	73 (43/59)
**Reader 2**			
Pseudocapsule			
T1WI/T2WI	27 (11/41)	50 (9/18)	34 (20/59)
T2*WI	61 (25/41)	67 (11/18)	61 (36/59)
SWI	88 (36/41)	67 (12/18)	81 (48/59)
Mosaic pattern			
T1WI/T2WI	(16/25)	(13/25)	(29/59)
T2*WI	(20/25)	(18/25)	(38/59)
SWI	25/25	(26/34)	(41/59)
Hemorrhage			
T1WI/T2WI	19 (8/43)	33 (6/18)	24 (14/59)
T2WI	86 (37/43)	39 (7/18)	73 (43/59)
SWI	98 (42/43)	67 (12/18)	92 (54/59)
Venous invasion			
T1WI/T2WI	23 (7/31)	57 (16/28)	39 (23/59)
T2*WI	84 (26/31)	61 (17/28)	73 (43/59)
SWI	90 (28/31)	75 (21/28)	83 (49/59)

**Table 3:** Overall sensitivity, specificity, and accuracy of morphologic features of hepatocellular carcinoma.

Pseudocapsule			
	SENS	SPEC	ACCUR
T1WI/T2WI	29	39	32
T2*WI	66	56	63
SWI	83	67	78
**Mosaic pattern**			
	**SENS**	**SPEC**	**ACCUR**
T1WI/T2WI	52	58	46
T2*WI	72	71	59
SWI	100	79	88
**Hemorrhage**			
	**SENS**	**SPEC**	**ACCUR**
T1WI/T2WI	16	69	31
T2*WI	88	56	80
SWI	95	81	92
**Venous invasion**			
	**SENS**	**SPEC**	**ACCUR**
T1WI/T2WI	23	61	41
T2*WI	77	68	73
SWI	90	82	86

All values in percent.

### Pseudocapsule

**Figure 1:**
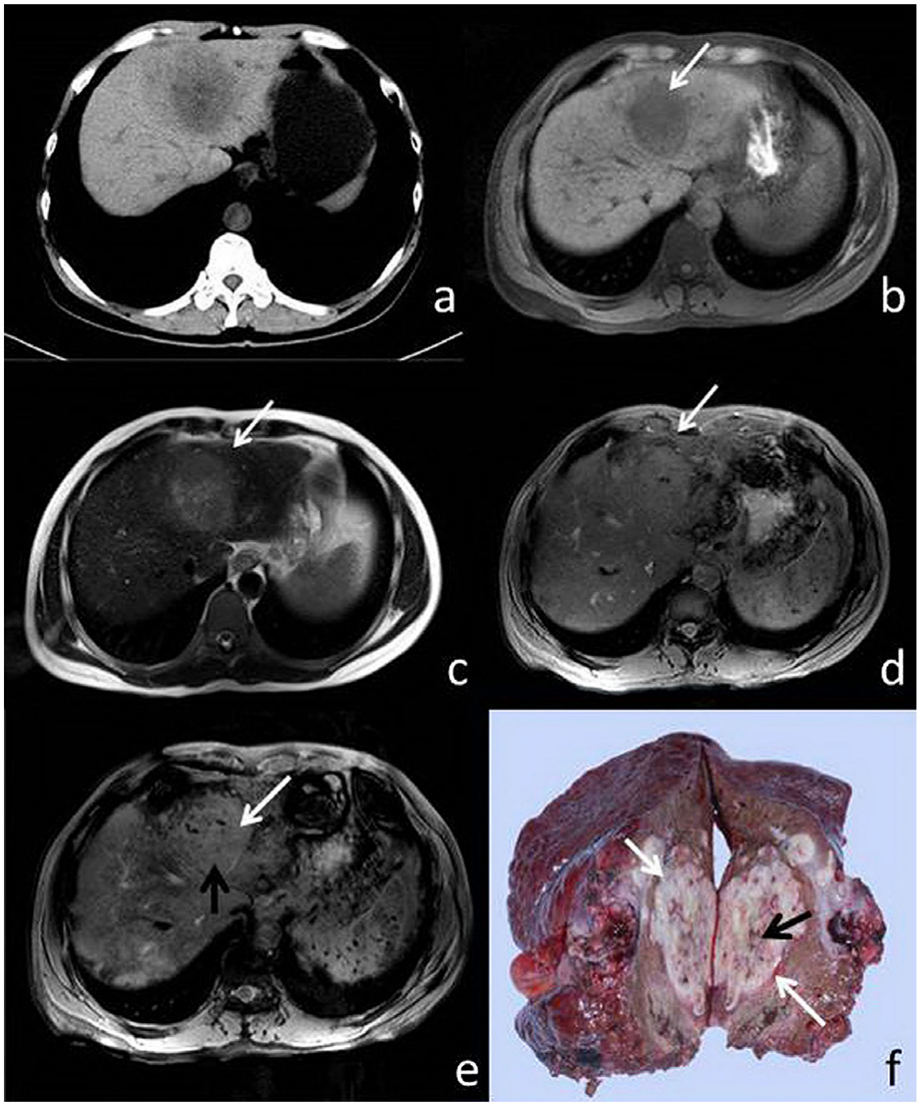
51-year-old male with HCC. (a) CT, (b) T1WI, (c) T2WI, (d) T2*, (e) SWI and (f) surgical specimen. The pseudocapsule is not perceptible on CT, T1W1, or T2WI (a–c), but is visible (white arrow) on both T2*WI and SWI (d, e). Both pseudocapsule and microhemorrhage (black arrow) are visible in the resected specimen (f) and SWI (e), and to a lesser extent, T2*WI (d).

**Figure 2:**
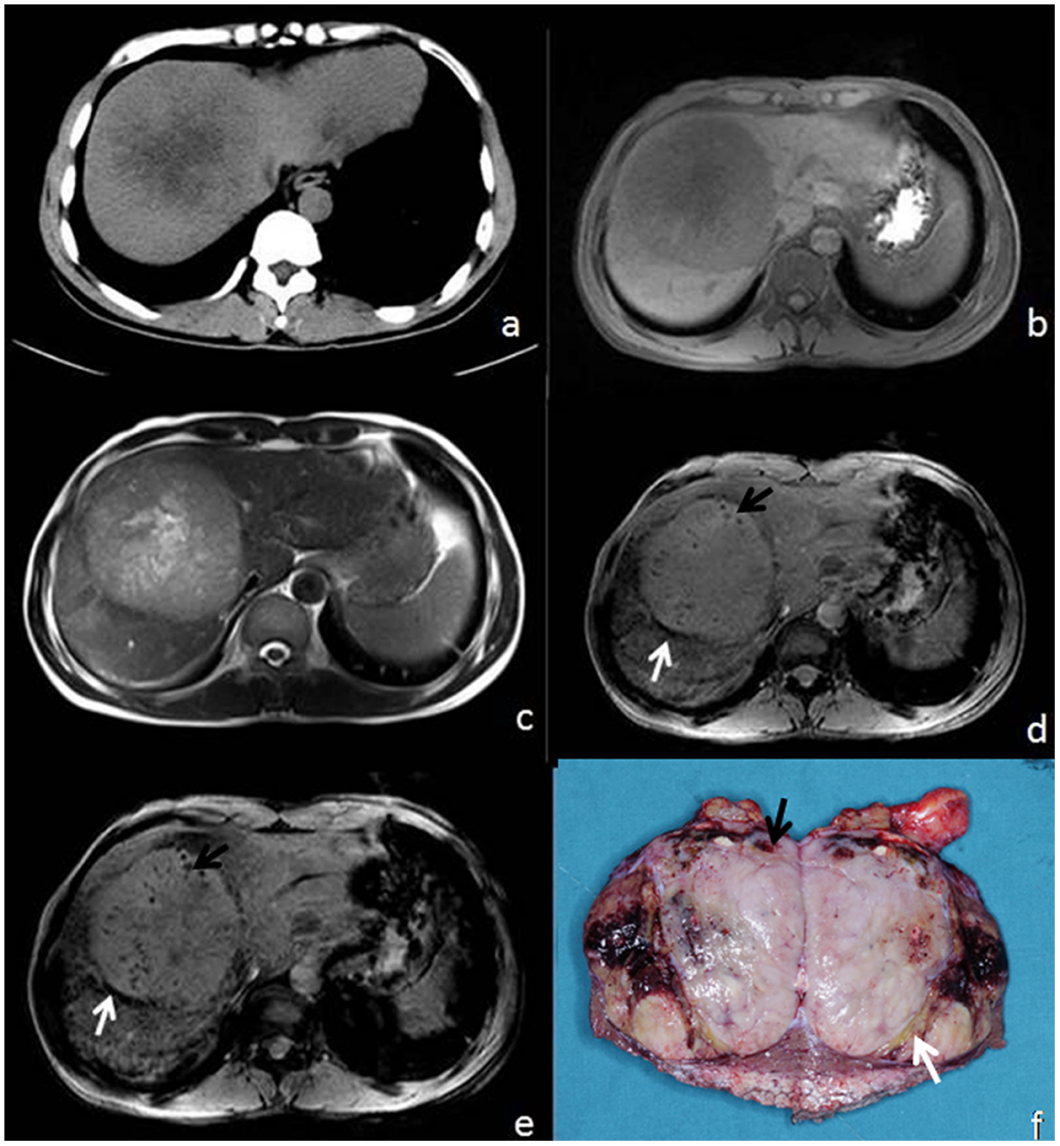
65-year-old male with HCC. (a) CT, (b) T1WI, (c) T2WI,(d) T2*, (e) SWI, and (f) surgical specimen. Pseudocapsule and foci of microhemorrhage are not visible on CT, T1WI, or T2WI (a–c), but the pseudocapsule (white arrow) and microbleeds (black arrow) are visible on both T2*WI (d) and SWI (e) as well as the surgical specimen (f). Here, SWI demonstrates the mosaic pattern of tumor heterogeneity (e).

On histopathology, pseudocapsules consist of prominent sinusoids and/or peritumoral fibrosis connecting to fibrosis of the Glisson sheath were observed at the tumor margin. The appearance of the pseudocapsule on imaging varied based on each modality. On T2*WI and SWI, the boundaries showed a low signal-intensity ribbon or a linear pattern (Figures 1, 2).

Histopathologically, a pseudocapsule was confirmed in 41 of 59 lesions (69%). On conventional T1WI and T2WI, a pseudocapsule was observed in 12 of 41 lesions (29%). On T2*WI, a pseudocapsule was observed in 27 of 41 lesions (66%). On SWI, a pseudocapsule was observed in 34 of 41 lesions (83%). There was no correlation between presence or absence of a tumor capsule and tumor size.

### Mosaic Pattern

**Figure 3:**
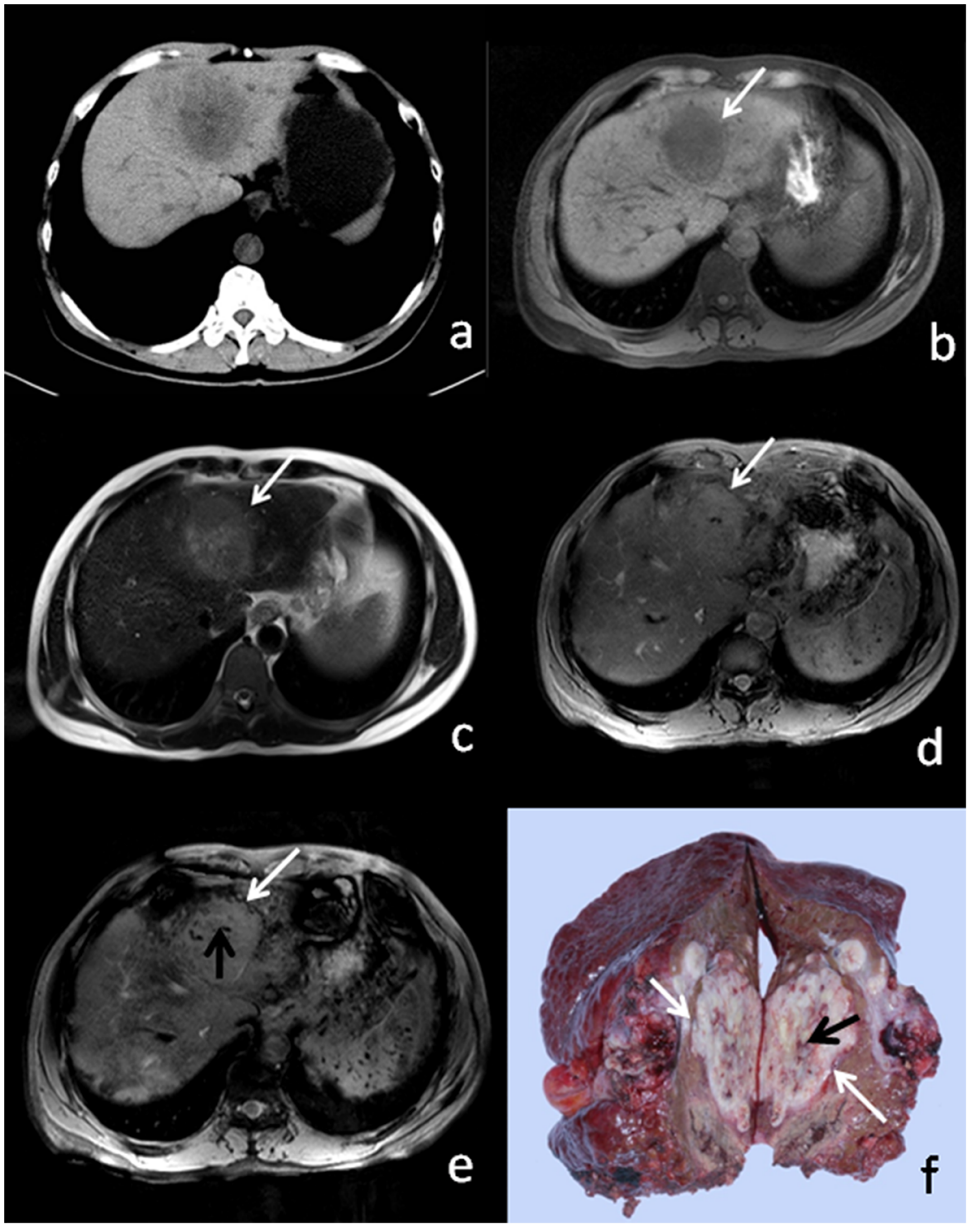
46-year-old male patient with HCC. (a) CT, (b) T1WI, (c) T2WI, and (d) T2* show no appreciable mosaic pattern or hemorrhagic foci. (e) SWI shows hypointense linear intratumoral structures (white arrow) and hemorrhagic foci (black arrow). (e) Resected surgical specimen shows internal microhemorrhage (white arrow) and linear structure (black arrow), which correlate with imaging findings.

Mosaic pattern was identified in 25 of 59 tumors (42%) histologically. On conventional T1WI and T2WI, mosaic pattern was depicted in 13/59 tumors (25%). On T2*WI, mosaic pattern was depicted in 18 of 59 lesions (31%). On SWI, a mosaic pattern was depicted in 25 of 59 lesions (42%), the same number detected histologically (Figures 2 and 3). Intratumoral linear-like structures of hypointensity were demonstrated on all sequences, though difference in the detection rate of mosaic patterns with T2-weighted images and SWI was statistically significant (*P*<0.01) compared to T1WI and T2*WI.

### Hemorrhage

Pathologically, hemorrhage was present within tumors in 43 of 59 lesions (73%). On T1WI and T2WI, hemorrhage was detected in 7 of 59 lesions (12%). On T2*WI, 38 of 59 lesions (64%) displayed hypointense foci indicating hemorrhage. On SWI, hemorrhage was visible in 41 of 59 lesions (69%). Two small HCCs were missed on SWI: one well-differentiated lesion with the greatest diameter 1.5 cm, and the other moderately-differentiated lesion with the greatest diameter 2.3 cm.

For qualitative analysis, using the grading system, SWI showed 11 lesions to be of grade 0 (no dot-like foci of hemorrhage), 15 lesions had a grade of 1, 23 lesions were graded 2, and 10 lesions graded 3. SWI revealed higher performance in detecting intratumoral hemorrhage (ITH) than CT, T1WI and T2WI (p<0.05). Only three new ITH were demonstrated on CT (Figure 3). Compared with T2*, SWI had slightly higher sensitivity. However, the difference was not statistically significant (p = 0.853).

### Vascular Invasion

**Figure 4:**
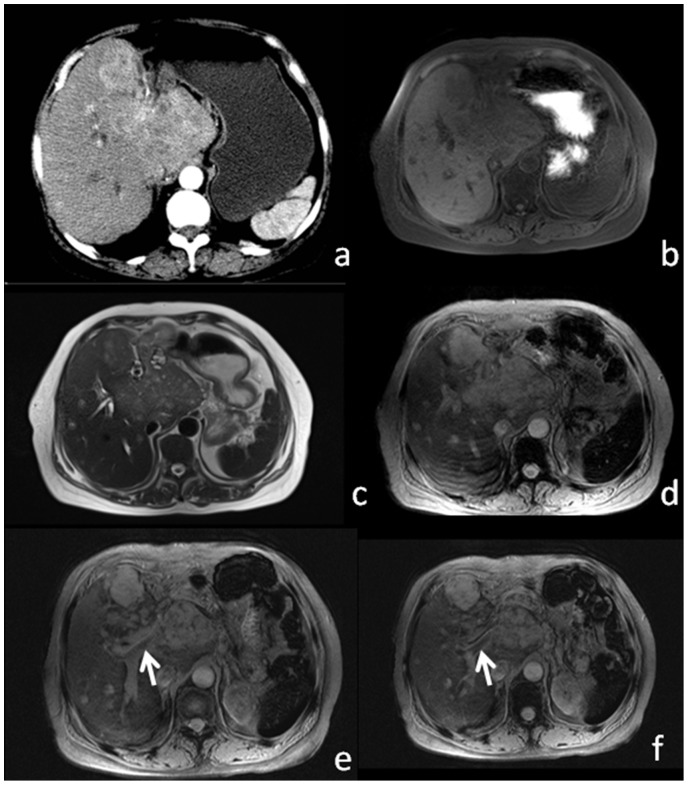
57-year-old male patient with large HCC. Enhanced CT (a) image shows patchy heterogeneous enhancement. T1WI (b) and T2WI (c) show substantial heterogeneous signal. T2*WI (d) and SWI (e) show a filling defect within the portal vein (white arrow), which corresponds to venous thrombus.

On histopathology, HCC can be observed inside hepatic and/or portal veins. On T2*WI and SWI, vascular invasion was characterized by a filling defect of the hyper-intense hepatic and/or portal vein lumens (Figure 4).

On histopathology, tumor invasion was present within the portal vein in 30 patients and within the hepatic vein in 1 patient. T1WI and T2WI demonstrated venous thrombi due to tumor invasion in 7 of 31 patients (23%). T2*WI demonstrated venous thrombi due to tumor invasion in 24 of 31 patients (77%). SWI demonstrated venous thrombi due to tumor invasion in 28 of 31 patients (90%).

## Discussion

In this study we compared the ability of multi-breath-hold 2D noncontrast SWI to identify HCC with that of conventional MRI (T1WI, T2WI) and T2*WI (Table 1). SWI was better at detecting both pseudocapsule and mosaic pattern, with a sensitivity of 83% for pseudocapsule and 100% for mosaic pattern compared to the T2*WI sensitivities of 66% and 72% (p = 0.03), and conventional MRI sensitivities of 29% and 52% for pseudocapsule and mosaic pattern, (p = 0.02). These results demonstrate that SWI is superior to T2, T2*, and noncontrast-enhanced T1 weighted MR images in characterizing HCC.

The presumed mechanism for the visualization of HCC with SWI is likely related to differential iron deposition within HCC and liver parenchyma [22]. Discrimination of the typically hypervascularized HCC nodules (malignant) from hypervascularized regenerative nodules (benign) is also presumably due to the fact that HCC no longer has the ability to uptake iron to the same degree as functional hepatic parenchyma [23]. SWI is more sensitive to changes in paramagnetic substances induced by a disease process (such as iron content, hemorrhage, calcification or changes in oxygenation level) than all other conventional MRI techniques including T2*-weighted because it uses phase information to enhance susceptibility effects in the images.

HCC tumor capsules, important for both lesion detection and surgical management, have an inner fibrous layer and an outer water-rich layer containing small vessels and bile ducts. This creates the appearance of a single hypointense ring on T1WI and a double ring with a hypointense inner layer with hyperintense outer layer on T2WI, or a single hypointense ring on T2WI for thin capsules due to paucity of vessels and bile ducts in the outer layer [3]. The pseudocapsule is also responsible for the hyperintense peripheral ring frequently seen on contrast-enhanced MRI [7]. Detection of the HCC pseudocapsule is important not only for the characterization of liver tumors, but also for clinical management. Pseudocapsules have rich microvascularity, and blood within these vessels has a low oxygen concentration, resulting in a phase difference between the vessels and the surrounding parenchyma. Therefore, SWI (which accentuates phase differences with increased image contrast) improves the conspicuity of the pseudocapsule compared to conventional T1WI and T2WI.

The mosaic pattern describes an internal lesion architecture seen histologically as well as with CT or MR imaging which, while variable, is formed from interspersed areas of growing tumor, regenerative liver tissue, and areas of necrosis [4], [5], [8]. This translates to an imaging appearance consisting of nodular foci (growing tumor or regenerative liver tissue), low-attenuation areas (necrosis or fibrosis), and internal septa (linear-like hypointensities and areas of differing intensity levels on conventional T1WI, T2WI, T2*WI, and SWI [4], [5], [8],

CT and MRI are the current standard diagnostic imaging modalities for the detection of HCC [24]. Pseudocapsule and mosaic pattern are two important features of HCC that are evident by histopathology and are detectable by imaging. In fact, one of the characteristic signs of HCC on CT is the ring sign formed by tumor pseudocapsule [21], [25]. MRI has been shown to be more reliable in the detection of HCC pseudocapsule, with MRI demonstrating the ring sign twice as often as CT [25]. The mosaic pattern is also a well-known characteristic sign of HCC on CT [8], though MRI has been shown to be superior to CT for detection of the mosaic pattern [9]. Comparing SWI to T1, T2, and T2* weighted MRI shows that SWI can give additional information during MRI characterization of HCC, opening up the potential for future applications of SWI in imaging for the evaluation of HCC. SWI is better than conventional noncontrast MRI for characterizing specific morphologic features of capsule, hemorrhage, macrovascular invasion, and identification of the mosaic pattern. The addition of SWI to routine hepatic MR imaging could lead to improved diagnostic specificity. The focus of future research could assess whether the overall MR imaging specificity for HCC is improved when read in conjunction with gadolinium-enhanced sequences.

SWI has been shown to improve the detection of siderotic nodules within the cirrhotic liver and better characterize intratumoral hemorrhages and venous vasculature in HCC [15], [16], [18]. Neovascularization increases during hepatocarcinogenesis, and hypervascularity is linked to histological grade. It has been reported that SWI is superior to the T1-weighted dynamic contrast-enhanced imaging for the detection of venous vasculature within HCC [18]. While both intratumoral blood products and blood within veins are paramagnetic, they can be detected and differentiated with SWI [19]. Similarly, SWI could permit the differentiation and detection of hypervascular lesions.

Many well-differentiated HCCs and high grade dysplastic nodules can have high signal intensity on T1-weighted images, which can be related to copper, iron, glycogen deposition, high protein/lipid content, and possibly the degree of cellular differentiation [26]. Dysplastic nodules are typically T2 hypointense, and well-differentiated HCCs can be isointense or (rarely) T2 hypointense compared to liver parenchyma; SWI may help to assess the different iron deposit of well-differentiated HCCs and dysplastic nodules.

This study has several limitations. First, despite being a prospective study, the sample size is limited. Second, gadolinium-enhanced MR sequences were not use as the imaging reference standard, and diffusion-weighted imaging was not part of our routine clinical imaging protocol. Third, histopathology analysis to identify other possible cellular elements (e.g., iron) possibly responsible for the visualization of HCC was not performed. Future studies are indicated to determine the full diagnostic performance of SWI in a larger patient population.

## Conclusions

In conclusion, our study demonstrates the feasibility of applying multi-breath-hold 2D SWI to HCC imaging, and shows that SWI is more sensitive and specific in identifying important morphologic features of HCC tumors without intravenous contrast, which is an important consideration in patients who cannot have gadolinium enhanced imaging due to lack of intravenous access, severe allergy, or severe renal functional impairment.
